# Detection of Anorectal and Cervicovaginal Chlamydia Trachomatis Infections following Azithromycin Treatment: Prospective Cohort Study with Multiple Time-Sequential Measures of rRNA, DNA, Quantitative Load and Symptoms

**DOI:** 10.1371/journal.pone.0081236

**Published:** 2013-11-20

**Authors:** Nicole H. T. M. Dukers-Muijrers, Arjen G. C. L. Speksnijder, Servaas A. Morré, Petra F. G. Wolffs, Marianne A. B. van der Sande, Antoinette A. T. P. Brink, Ingrid V. F. van den Broek, Marita I. L. S. Werner, Christian J. P. A. Hoebe

**Affiliations:** 1 Department of Sexual Health, Infectious Diseases, and Environmental Health,South Limburg Public Health Service, Geleen, The Netherlands; 2 Department of Medical Microbiology, School of Public Health and Primary Care (CAPHRI), Maastricht University Medical Center, Maastricht, The Netherlands; 3 Public Health Laboratory, Health Service Amsterdam, Amsterdam, The Netherlands; 4 VU University Medical Center, Medical Microbiology and Infection Control, Amsterdam, The Netherlands; 5 Institute of Public Health Genomics, Department of Genetics and Cell Biology, Research Institute-GROW, Faculty of Health, Medicine and Life Sciences, University of Maastricht, Maastricht, The Netherlands; 6 Epidemiology and Surveillance, Centre for Infectious Diseases Control, National Institute for Public Health and the Environment, Bilthoven, The Netherlands; 7 Julius Center, University Medical Centre Utrecht, Utrecht, The Netherlands; University Hospital San Giovanni Battista di Torino, Italy

## Abstract

**Background:**

Determination of *Chlamydia trachomatis* (Ct) treatment success is hampered by current assessment methods, which involve a single post-treatment measurement only. Therefore, we evaluated Ct detection by applying multiple laboratory measures on time-sequential post-treatment samples.

**Methods:**

A prospective cohort study was established with azithromycin-treated (1000 mg) Ct patients (44 cervicovaginal and 15 anorectal cases). Each patient provided 18 self-taken samples pre-treatment and for 8 weeks post-treatment (response: 96%; 1,016 samples). Samples were tested for 16S rRNA (TMA), bacterial load (quantitative PCR; Chlamydia plasmid DNA) and type (serovar and multilocus sequence typing). Covariates (including behavior, pre-treatment load, anatomic site, symptoms, age, and menstruation) were tested for their potential association with positivity and load at 3–8 weeks using regression analyses controlling for repeated measures.

**Findings:**

By day 9, Ct positivity decreased to 20% and the median load to 0.3 inclusion-forming units (IFU) per ml (pre-treatment: 170 IFU/ml). Of the 35 cases who reported no sex, sex with a treated partner or safe sex with a new partner, 40% had detection, i.e. one or more positive samples from 3–8 weeks (same Ct type over time), indicating possible antimicrobial treatment failure. Cases showed intermittent positive detection and the number of positive samples was higher in anorectal cases than in cervicovaginal cases. The highest observed bacterial load between 3–8 weeks post-treatment was 313 IFU/ml, yet the majority (65%) of positive samples showed a load of ≤2 IFU/ml. Pre-treatment load was found to be associated with later load in anorectal cases.

**Conclusions:**

A single test at 3–8 weeks post-treatment frequently misses Ct. Detection reveals intermittent low loads, with an unknown risk of later complications or transmission. These findings warrant critical re-evaluation of the clinical management of single dose azithromycin-treated Ct patients and fuel the debate on defining treatment failure. Clinicaltrials.gov Identifier: NCT01448876.

## Introduction

The current clinical determination of treatment success in *Chlamydia trachomatis* (Ct) infections is based on antimicrobial assessment, i.e., a test-of-cure, in a treated patient who is considered at low sexual re-exposure risk [1]. Usually, a single nucleic acid amplification test (NAAT) is applied after 3 weeks and when positive, taken as antimicrobial treatment failure. However, testing for cure is not routinely recommended because it has major shortcomings [2]–[4]. A single positive NAAT may reflect a re-infection, even when the sexual re-exposure risk is considered low. It also may originate from non-viable Chlamydia DNA. Furthermore, a negative NAAT does not exclude treatment failure, as persistent infections produce intermittent negative post-treatment results [5]–[6]. Nonetheless, substantial antimicrobial treatment failure rates in patients with an assumed low re-infection risk have been reported for azithromycin, the first-line treatment for Ct [5], [7]. Rates range from 5–14% in genital Ct infection [8]–[14] and from 6–21% in asymptomatic anorectal infection [15]–[17]. While failure in these studies may be misclassified due to the aforementioned shortcomings and the clinical implications of antimicrobial detection are not always clear, another problem is that the underlying mechanisms responsible for treatment failure are poorly understood. Horner recently postulated that single-dose antibiotic exposure might be too short-lived given the complex Ct life cycle [18]. Non-replicating (non-infectious) elementary Ct bodies may survive treatment and revert to the replicating (infectious) reticulate body when antibiotic levels decrease [19]. Furthermore, azithromycin tissue concentrations are unknown in anorectal Ct cases and might be too low after oral antibiotics [8], [17]. Heterotypic resistance is suggested to play a role, especially when the pre-treatment bacterial load is high [18]. However, there is currently no solid evidence of in vivo antibiotic resistance for genital or anorectal Ct infection, and routine laboratory resistance tests are lacking. Hence, many issues involving the extent, causes and clinical implications of persistent Ct detection after treatment remain unsolved. As a pragmatic solution for improving patient management, an increased role for test-of-cure practice by single NAAT testing is advocated [7], [18]. However, such practice is unlikely to adequately address its aim given the above-mentioned uncertainties regarding the meaning of a single positive or negative NAAT result [2]–[4]. Therefore, there is a need for treatment evaluation studies with more intensive follow-up and the use of several complementary laboratory assays.

Here, we present Ct assessment in a prospective cohort of azithromycin-treated patients followed for 2 months post-treatment, providing multiple time-sequential measures of bacterial load, symptoms, behavioral and microbiologic re-infection markers, i.e., sequence typing, to increase our understanding of treatment failure, bacterial detection after treatment and clinical treating and testing implications.

## Methods

### Ethics statement

Participants provided written consent to participate in this study. The study, including the consent procedure, was approved by the Medical Ethics Committee at the VU University of Amsterdam (2009/154, CCMO The Hague: NL28609.029.09). Clinicaltrials.gov Identifier: NCT01448876.

### Study population and procedures

**Figure 1:**
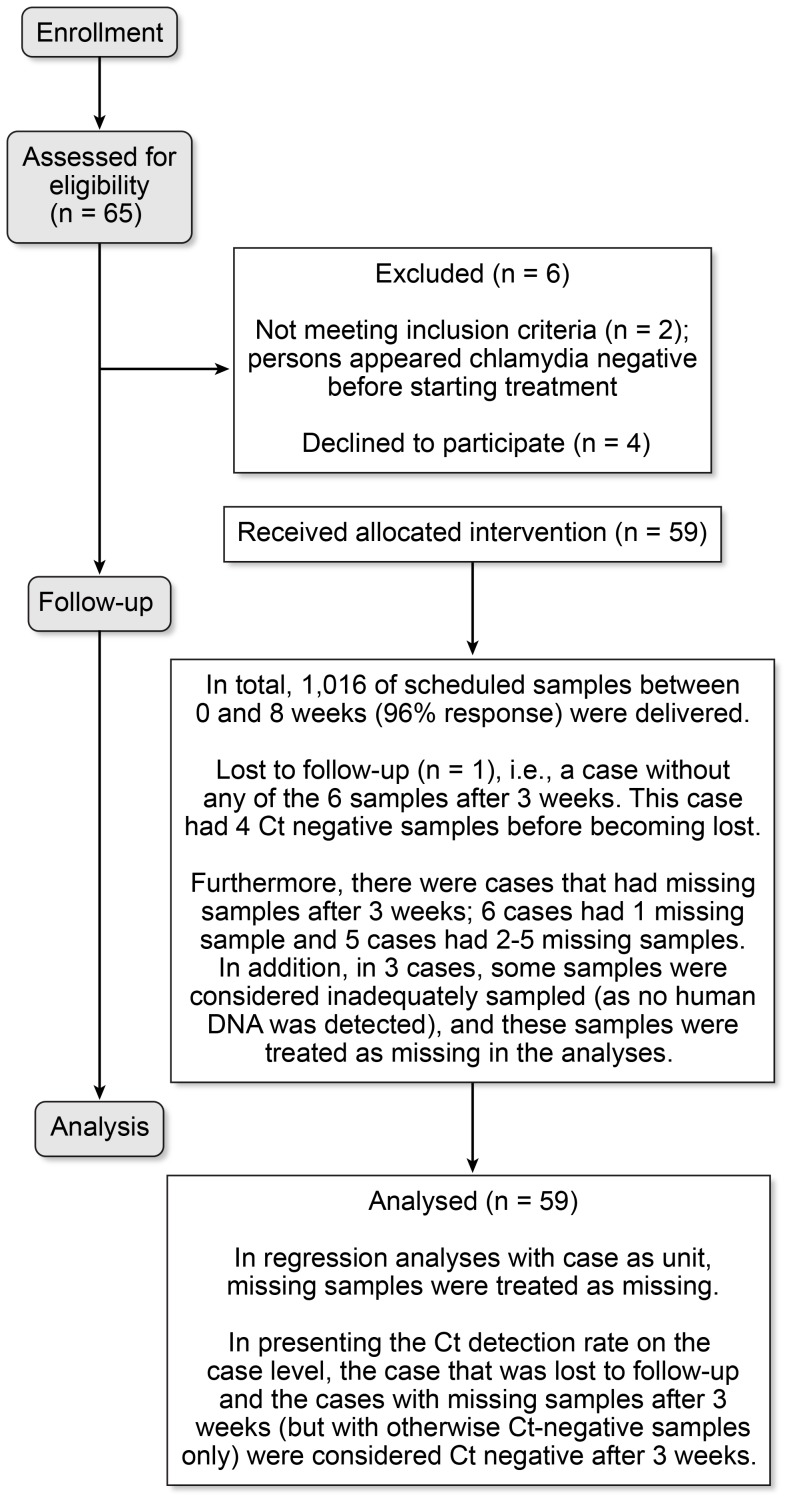
Flow diagram participants from recruitment to analyses.

**Figure 2:**
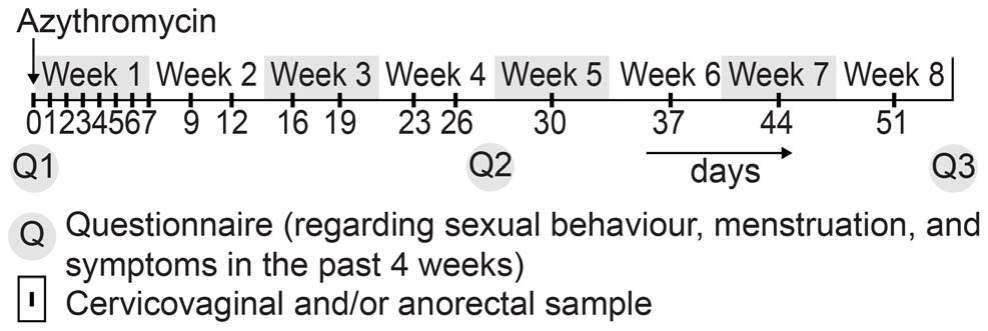
Study sampling frame for anorectal and cervicovaginal swabs and questionnaires.

A convenience sample of Ct-diagnosed patients was recruited at our outpatient STD clinic (South Limburg, the Netherlands) between June 2009 and June 2010. Patients were eligible when they were diagnosed with anorectal and/or cervicovaginal Ct, were HIV-negative, aged ≥18 years, did not use antibiotics in the week before diagnosis, did not use immunosuppressive medication, were not pregnant, understood the Dutch language and did not self-identify as a commercial sex worker or a swinger [20]. In total, 52 patients participated, with 59 Ct infections (Fig. 1). All patients were negative for *Neisseria gonorrhea*, syphilis, hepatitis B and Chlamydia serovar L1-3b (Lymphogranuloma venereum) [4]. At day 0 (study intake), administration of a single oral azithromycin dose (1000 mg) was observed. Usual care was given by recommending abstinence or safe sex for 1 week and treating steady partners with a single dose of 1000 mg azithromycin [1], [2]. Self-taken cervicovaginal and/or anorectal swabs were provided at 18 pre-defined time-points over an 8-week period at day 0 (pre-treatment) and days 1–51 (post-treatment) (Fig. 2). After sampling, a swab was placed directly into the test medium (including RNA stabilizers), mailed to the laboratory and stored at −20°C until further processing. At intake and at the end of weeks 4 and 8, participants completed a self-administered questionnaire on demographics, menstruation, sexual behaviors and symptoms over the past 4 weeks. To increase response, participants were contacted on a weekly basis by the study nurse and at the end of weeks 4 and 8, participants received a small monetary incentive (coupon 15 euro).

### Laboratory testing and interpretation

#### NAAT testing and quantification of Chlamydia load

Ct screening (16S rRNA) was performed with the Aptima system (Aptima CT, Gen-Probe, San Diego, USA) using 400 µl of prepared sample of a total volume of 2.9 ml. For DNA testing, a real-time PCR targeting the cryptic plasmid was performed using 10 µl of prepared sample (in-house PCR; Amsterdam, The Netherlands) [21]. Samples that tested negative for DNA were retested. DNA isolates were stored at −20°C until further use. The Ct-DNA load was expressed as inclusion-forming units per ml. (IFU/ml) based on defined serial dilutions of Ct cultured in human cells with over >90% infected HeLa cells of 100 IFU to 0.001 IFU, also taking into account DNA from non-viable Ct particles [22].

#### Positive samples

A sample was considered positive when rRNA and/or DNA were detected. Nearly all Ct DNA-positive samples were also rRNA-positive (395/407; 97%); the DNA load in the few rRNA-negative cervicovaginal samples ranged from 0.2 to 16 IFU/ml (median: 0.7 IFU/ml). Some of the rRNA positive samples were DNA-negative (95/490; 19%).

#### Sequence typing

In cases with DNA isolates from 23–51 days post-treatment, serovar determination was performed by OMP1 gene sequencing with confirmatory quantitative PCR DNA screening using a reverse hybridization assay [23] (Labo Bio-medical Products, Rijswijk, The Netherlands). The subjects who reported unsafe sex with an untreated partner were further typed by multilocus sequence typing as previously described [24]. Sequence typing was performed on the intake sample, the last positive sample, and when present, another positive sample mid-follow-up.

#### Sampling errors

**Figure 3:**
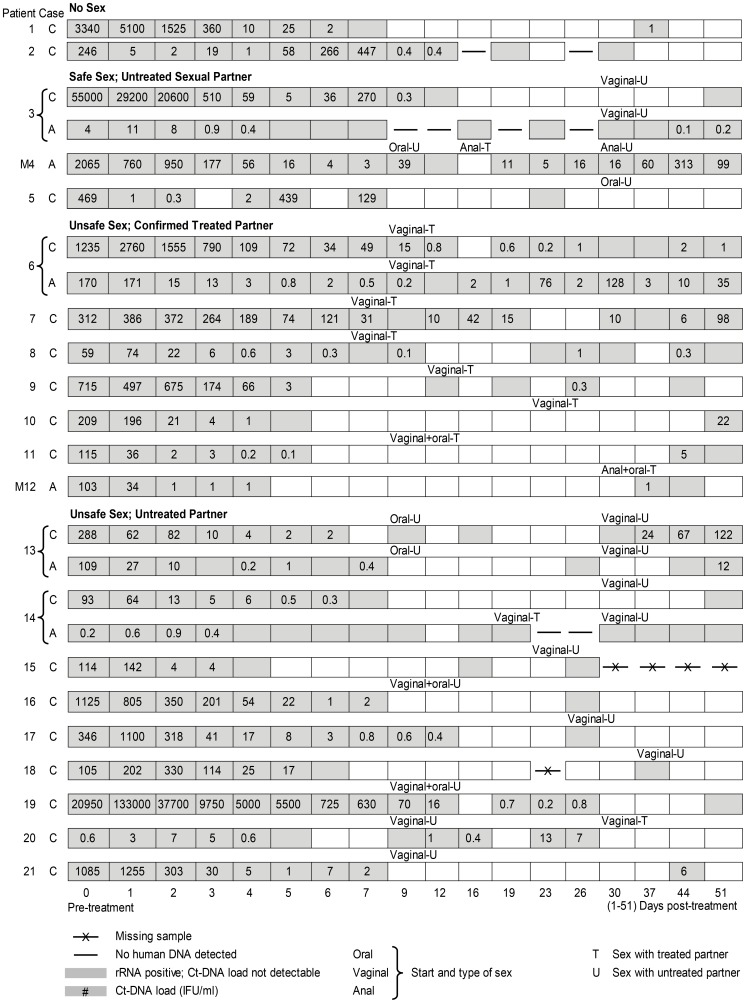
*Chlamydia trachomatis* positivity and load among cases of cervicovaginal (C) and anorectal (A) infections in female and male (M) patients by sexual behavior.

To rule out the possibility that a negative result was due to inadequate self-sampling, negative samples were re-tested for human DNA (HLA). In total, 8 of the 514 negative samples (1.6%) from 3 cases did not contain human DNA (Fig. 3, cases 2C, 3A and 14A); these 8 samples were considered missing, resulting in 1,008 samples in the statistical analyses.

### Definitions per case

A case is a Ct infection. A patient may contribute 1 or 2 cases, i.e. anorectal and/or cervicovaginal. Each case delivered 6 samples in the period between 23–51 days post-treatment (Fig. 2). A case was defined to have Ct detection when one or more of these 6 samples were Ct positive.

Based on self-reported behavior during follow-up, each case was assigned to 1 of 5 hierarchically constructed groups from low to high sexual re-exposure risk: (1) no sex or sex with a tested negative partner only, (2) sex with a confirmed treated partner only, (3) safe sex (always using condoms in anal and vaginal sex) with a new (considered untreated) sex partner, (4) unsafe sex (not always using condoms in anal or vaginal sex) with a new sex partner and (5) unknown sexual behavior.

The cases that were assigned to categories 4 or 5 were considered at high risk for re-infection [1]. A case with different Ct types over time was considered a proven re-infection. All other cases, i.e. the cases that did not show a different Ct type over time and also were assigned to low sexual re-exposure risk (categories 1, 2 or 3), were considered to have low re-infection risk. In such cases, Ct detection was taken to reflect possible antimicrobial failure.

### Statistical analyses

Descriptive statistics were performed to present the positivity rates and median load for each assessed time-point. Regression analyses were performed that controlled for repeated measurements by clustering by case to assess the association between covariates and Ct. Therefore, covariates were assessed for (1) positivity (using generalized estimating equations for logistic regression) and (2) log-transformed quantitative load (using mixed-effects models) where rRNA-positive but DNA-negative samples were considered positive with 0 IFU/ml Ct-DNA load. Two time periods were particularly emphasized: the first 9 days (routine care recommends safe or no sex in the first week), and the period from 23 to 51 days post-treatment in accordance with previous treatment failure studies and current test-of-cure practice with assessment after 3 weeks [1], [2], [8]–[17]. Covariates under study included fixed covariates [pre-treatment cervicovaginal symptoms (yes/no; women only); pre-treatment load (cutoff: anatomic site specific 75% quartile); age (cut-off: median); sex; Ct diagnosis history; sexual re-exposure risk (high risk categories 4 and 5 versus low risk categories 1–3); and anatomic infection site], and time-dependent covariates [days since treatment (continuous), menstruation (yes/no; women only) and anatomic site-specific current symptoms (yes/no)]. Estimates were adjusted for sexual re-exposure risk and anatomic infection site as a priori these were considered to be possible confounders. All mentioned covariates were also checked for possible interaction with sexual re-exposure risk or the anatomic infection site. In analyses missing samples were not included.

When presenting the detection rate, the cases with samples that were scheduled between 23–51 days missing (while all tested samples in this time period were negative) were taken as having no Ct detection after 3 weeks. In cases with detection, a Poisson regression analysis was used to assess the association between the number of positive samples and the listed fixed covariates. As this study was explorative in nature, a power calculation was not performed a priori. Analyses were performed using SPSS package version 21 (IBM, Inc.).

## Results

In total, 59 cases were contributed by 52 Caucasian patients (Table 1). Cases included cervicovaginal Ct in 44 women and 15 anorectal Ct in 8 men who had sex with men and in 7 women (who also participated with cervicovaginal infections). All women used hormonal contraception. Pre-treatment, STI-related symptoms were reported by 1 anorectal and 27 cervicovaginal cases. Overall, 1,016 samples (96% response) were delivered according to schedule and laboratory tested.

**Table 1:** Clinical and microbiological characteristics of the 59 *Chlamydia trachomatis* infections.

	Cervicovaginal	Anorectal	
	N = 44	N = 15	*P-*
	% (n)	% (n)	*value*
Median age in years (IQR)	23 [21]–[25]	23 [21–28]	
Female^a^	100% (44)	46.7% (∧7)	
Self-reported history of Ct			
Not tested before	52.3% (23)	53.3% (8)	
Tested Ct-negative	25.0% (11)	33.3% (5)	
Tested Ct-positive	22.7% (10)	13.3% (2)	
Self-reported symptoms			***
No	25.0% (11)^b^	73.3% (11)^a^	
Pre-treatment only	22.7% (10)^b^	0	
Pre-treatment and during follow-up	38.6% (17)^b^	6.7% (1)^c^	
During follow-up only	13.6% (6)^b^	20.0% (3)^c^	
RNA/DNA positivity and Ct-DNA load			
Day 0 (Pre-treatment)			
Ct-positive cases	100% (44)	100% (15)	
Load (median IFU/ml [IQR])	279 [96–1299]	33 [4–159]	**
Day 9 (Post-treatment)			
Ct-positive cases	28.6% (12)	20.0% (3)	
Load (median IFU/ml [IQR])	0.4 [0–13]	0.6^d^	

Abbreviations: IQR: Interquartile range; Ct Chlamydia trachomatis.

**P<0.01;

***p<0.001.

a 7 women participated with both cervicovaginal and anorectal infections.

b dysuria; irregular menstruation; lower abdominal pain; pain during intercourse; vaginal discharge.

c anal discharge; anal blood loss during/after intercourse; anal pain during/after intercourse.

d no IQR calculated due to insufficient number of cases.

### Positivity and load from 0 to 8 weeks post-treatment

**Figure 4:**
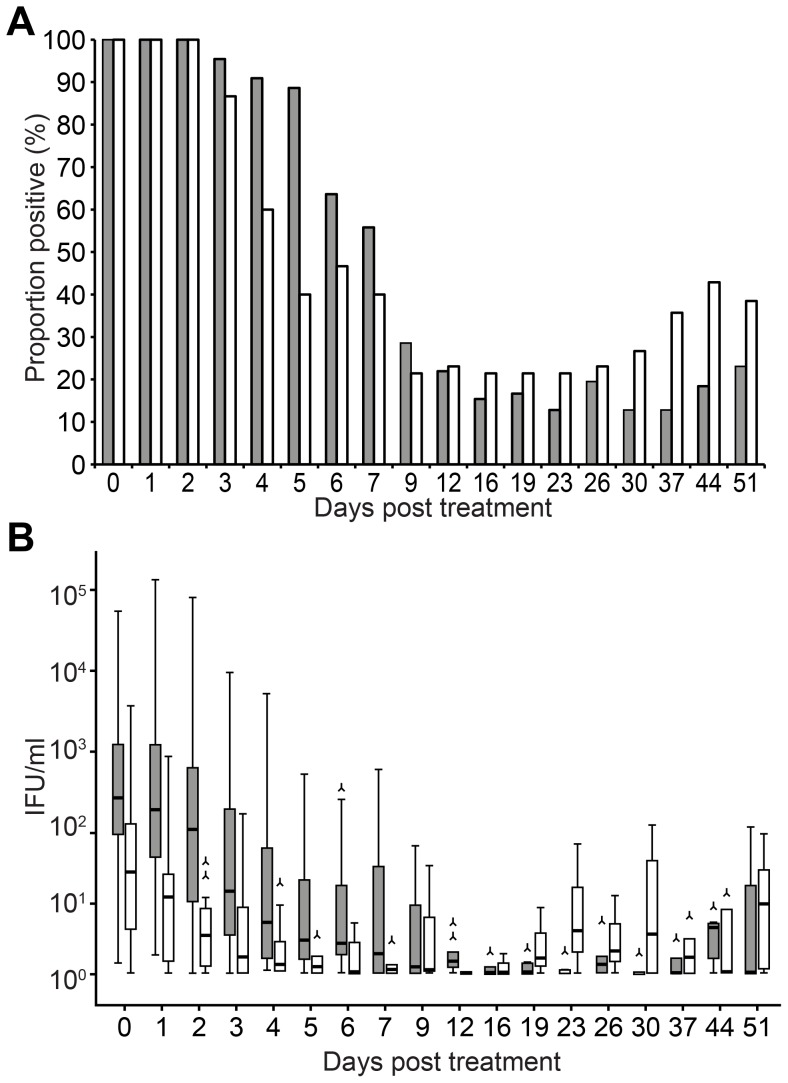
*Chlamydia trachomatis* positivity and load pre-treatment and 1–51 days post-azithromycin treatment; 15 anorectal (white bars) and 44 cervicovaginal (gray/black bars) infections. a. Proportion positive (%) (Chlamydial rRNA and/or DNA). b. Quantitative Chlamydial DNA load (boxplot; IFU/ml).

The sample proportion testing positive for Ct (positivity) decreased from 100% pre-treatment to 27% (15/56) by day 9. With each day of sampling, the odds of having a positive sample was reduced by 9%, as reflected in an Odds Ratio (OR) for each subsequent day of sampling of 0.91 per day (95% Confidence Interval (CI): 0.90–0.92). The overall median load decreased from 170 IFU/ml pre-treatment to 0.3 IFU/ml by day 9 (log load/day; −0.24, 95% CI: −0.28; −0.20) (Table 1) Positivity was 15% at day 23 and 27% at day 51 (Fig. 4 displays positivity by anatomic site). Positivity and load (marginally) increased from weeks 3–8 (Table 2).

**Table 2:** Case-level analyses on the associations between potential covariates and *Chlamydia trachomatis*-positive samples (positivity) and load from days 23–51 after treatment.

	Positivity, Days 23–51	Load, Days 23–51
	OR 95% CI a	Difference log load
		(IFU/ml) 95% CI a
Time post treatment (per day)	1.004 (1.00;1.01) #	0.01 (0.001;0.02) *
Anorectal case	1.16 (0.92;1.45)	0.19 (−0.22;0.61)
Age <23 years	1.06 (0.90;1.24)	−0.05 (−0.41;0.32)
History of Ct diagnosis	0.96 (0.81;1.14)	0.14 (−0.39;0.69)
Pre-treatment load >75% percentile b	1.01 (0.83;1.23)	Anorectal: 0.99 (0.04;2.02)^c^ *
		Cervicovaginal: −0.16 (−0.75;0.42)^c^
Current symptoms d ^,^ e	1.02 (0.89;1.16)	−0.20 (−0.47;0.07)
Pre-treatment symptoms d ^,^ f	0.94 (0.80;1.11)	−0.31 (−0.71; −0.09)
Current menstruation f	0.96 (0.86;1.07)	0.03 (−0.33;0.40)
Sexual re-exposure risk: unsafe sex	0.99 (0.69;1.09)	−0.07 (−0.45;0.32)
with a new partner/unknown behavior		

Abbreviations: OR: Odds Ratio; CI: Confidence Interval; IFU/ml: Inclusion-forming units per milliliter.

#: P = 0.06,

*P<0.05; tested using generalized estimating equations (positivity) or mixed models (load).

a estimates adjusted for potential confounding by sexual re-exposure risk, anatomic site, and repeated measurements; in models with bacterial load as outcome, anatomic site was included as an effect-modifier as the interaction-term was statistically significant.

b >159 IFU/ml for anorectal cases and >1299 IFU/ml for cervicovaginal cases.

c presented separately for anatomic sites due to significant interactions between pre-treatment load and site (P = 0.008).

d dysuria; irregular menstruation; lower abdominal pain; pain during intercourse; vaginal discharge.

e anal discharge; anal blood loss during/after intercourse; anal pain during/after intercourse.

f only evaluated for cervicovaginal cases.

Pre-treatment, the bacterial load was higher in anorectal cases than in cervicovaginal cases. However, load did not significantly differ between anatomic sites by day 9 (Table 1) or after 3 weeks (Table 2).

### Cases' times to first negative and subsequent positive samples

At the case level, the first negative sample observed was within 9 days after treatment for 78% (46/59) of cases (see Fig. 3 for some case-time examples). The time to first negative sample ranged from 2 to 21 days, and 2 cases never had negative samples; in 75% of cases, the load in the sample preceding these first negative samples was less than 2 IFU/ml. However, in 63% (36/57) of cases with a negative sample, their first negative sample was followed (range 1–45 days) by subsequent positive sample(s).

### Detection and load after 3 weeks and covariates

In total, 25 of the 59 cases had a positive sample between 3–8 weeks; 56% (14/25) of these cases also had a negative sample within 9 days. Sequence typing revealed the same type at intake and follow-up for each case. The load observed between 3–8 weeks was lower than the pre-treatment load (except for case 20C, Fig. 3). Of all positive samples after 3 weeks (n = 65), 65% (n = 42) had a load ≤2 IFU/ml.

In the 35 cases with low re-infection risk (i.e., who had the same Ct type over time and reported low sexual re-exposure risk), 40% had at least one positive sample between 3–8 weeks. In cases that reported high re-infection risk (unsafe sex with a new partner or unknown behavior), 46% had at least one positive sample (Table 3). Sexual re-exposure risk was not associated with positivity or with load; anatomic site was not associated with positivity (Table 2).

**Table 3:** Treated cases with *Chlamydia trachomatis* detection after 3 weeks, i.e. having at least 1 positive sample out of 6 samples taken between 23–51 days post-treatment.

	No sex/sex with Chlamydia-negative partner/safe sex			Unsafe sex with untreated partner/		
	with untreated partner/unsafe sex with treated partner			unknown behavior		
	Cervicovaginal	Anorectal	Total	Cervicovaginal	Anorectal	Total
*N cases*	*24*	*11*	*35*	*20*	*4*	*24*
Detection; % (n)	42% (10/24)	36% (4/11)	40% (14/35)	45% (9/20)	50% (2/4)	46% (11/24)
Only cases with no sex/sex with negative partner; % (n)	20% (2/10)	0% (0/4)	14% (2/14)			
Only cases with safe sex with untreated partner; % (n)	67% (2/3)	40% (2/5)	50% (4/8)			
Only cases with sex with treated partner; % (n)	55% (6/11)	100% (2/2)	62% (8/13)			
Only cases with unsafe sex with untreated partner; % (n)				53% (9/17)	67% (2/3)	55% (11/20)
Only cases with unknown behavior; % (n)				0% (0/3)	0% (0/1)	0% (0/4)
Cases with 4–6 positive samples, % (n)	13% (3/24)	27% (3/11)	17% (6/35)	5% (1/20)	25% (1/4)	8% (2/24)
Cases with 1–3 positive samples, % (n)	29% (7/24)	9% (1/11)	23% (8/35)	40% (8/20)	25% (1/4)	38% (9/24)
Median number of positive samples (min 1–max 6) per case	1.5 (1–6)	5.5 (2–6)		1 (1–4)	3.5 (3–4)	

Abbreviations: IFU/ml: Inclusion-forming units per milliliter.

In analyses controlling for repeated measurements and sexual re-exposure risk and including interactions with anatomic sites, a high pre-treatment load was associated with a later load in anorectal cases but not in cervicovaginal cases (Table 2).

### Case-time-patterns in Ct test results and load

In total, 65 (20.4%) of all samples taken between 3 and 8 weeks tested positive. Positive samples originated from the 25 cases with Ct detection of whom 21 had at least one Ct negative sample after 3 weeks. Of the cases with Ct detection, the number of positive samples was 2.1 times (95% CI: 1.2–3.6; p = 0.009) higher in anorectal infections (estimated mean: 4 positive samples) than in cervicovaginal infections (estimated mean: 2 positive samples). No other fixed covariates under study were associated with the number of positive samples.

Fig. 3 displays on an individual case-time level the Ct test results and loads. For example, of the cases with low re-infection risk and 4 to 6 positive samples, some had loads ≤2 IFU/ml (Fig. 3, case 3A, 6C, 8C), while others showed higher loads (Fig. 3, case 4A, 6A, 7C) from 5 IFU/ml to 313 IFU/ml. Of the cases with low re-infection risk and between one and 3 samples positive, the the highest observed load was 22 IFU/ml.

## Discussion

Frequent time-sequential Ct assessments, quantitative bacterial load and symptoms revealed a high Ct detection rate 3–8 weeks after azithromycin treatment in uncomplicated anorectal and cervicovaginal infections.

To understand whether the observed antimicrobial Ct detection reflects possible treatment failure, re-infection should be ruled out. Usually, this is achieved with exclusions or adjustments in analyses for cases that reported high-risk sexual behavior, i.e., unsafe sex with an untreated partner [1], [8]–[17]. By excluding high-risk patients, our observed 40% detection rate exceeded previous reports and was similar in both anatomic sites [8]–[17]. Detection was revealed as highly intermittent. Only 3 or less (out of 6) samples tested positive in the large majority of the cervicovaginal cases and in a minority of the anorectal cases. Intermittent detection will partly be missed in single-point testing (i.e., cases will test negative). This suggests that by not including multiple repeated testing, previous studies have underestimated Ct detection after treatment in both anatomic sites, especially in cervicovaginal infections.

Some cases may still be attributed to re-infection when patients (1) have falsely reported not having had unsafe sex with an untreated partner or (2) have been re-infected by their treated sex partner. While both possibilities represent general limitations for treatment failure studies, the latter represents a novel hypothesis with clinical implications. This hypothesis is corroborated by our strikingly high detection rates (62%) in cases that reported having had sex only with an azithromycin-treated partner, i.e., either by directly observed treatment or by expedited partner treatment, including (phone) confirmation by the study nurse. As a treated partner is also likely to have positive post-treatment (intermittent) Ct detection, partners may continue to transmit Ct to each other, even when Ct concentrations are low. The possibility that this partner is re-infected by a third partner cannot be ruled out either, but it is highly unlikely to explain the high detection rates. Further, some cases now attributed to possible re-infection (i.e., all cases with detection and also reporting unsafe sex with a new partner) may actually represent treatment failure. For these cases to all represent re-infections would require their new partners to have a Ct positivity rate of 55%, i.e., the detection rate in the index cases. There was one patient (Fig. 3, case 14A) reporting unsafe vaginal sex but no anal sex, who had almost exclusively positive anorectal samples in her complete 8 weeks of follow-up. Most of the cases reporting unsafe sex with an untreated partner presented with few positive tests, i.e. at most reflected transient re-infections. Only 1 case (Fig. 3, case 13C) suggested an established re-infection after unsafe sex, showing increasing bacterial loads. Finally, self-infections from one anatomic site to another cannot be ruled out in women with both anorectal and cervicovaginal detection (Fig. 3, patients 3, 6, 13, and 14). Unfortunately, data did not allow for sensitivity analyses to explore this further, as excluding these women would result in only few anorectal infections available for analyses and as anorectal infections could not be ruled out in the patients who were not tested anorectally. In spite that there may be bias due to possible case misclassifications, the post-treatment detection rate was likely higher than is generally assumed based on single antimicrobial assessment [8]–[17].

The cases presented in the current study showed a variation of detection patterns and load levels. NAAT tests are highly sensitive and may detect low Ct concentrations. Indeed, many positive samples between 3–8 weeks had low Ct DNA loads (e.g., two-thirds of cases exhibited ≤2 IFU/ml) and were often preceded and/or followed by negative or low-load samples. Such intermittent and consistent low-load detection may reflect a low-level infection that is near the detection limits of the tests. Still, residual non-viable Chlamydial infection cannot be ruled out for these low-load cases, and further study is required to establish whether non-viable Ct can be detected up to 8 weeks after treatment. There were 3 cases with low re-infection risk that showed consistent detection with higher and upward load trends (Fig. 3, case 4A, 6A, 7C). Such patterns may reflect a small resistant population reemergence. Heterotypic resistance may occur at high pre-treatment load levels; this type of resistance has also been hypothesized to present with Chlamydial aberrancy [19], [25]. The cases in question had pre-treatment load levels that exceeded the median levels (Table 1), and overall regression analyses revealed high pre-treatment load as a higher load predictor in anorectal cases. Notably, our findings were limited by the comparatively low number of anorectal cases, decreasing our capacity to detect differences between anatomic sites. Further, this study did not include genital Ct in men, and findings may not be applicable to this population. While the cases presented here may indeed reflect treatment failure, the salient point is that the clinical relevance of failure as defined by antimicrobial detection is largely unknown and not studied. For example, what are the clinical implications (e.g., transmission, morbidity, treatment and screening practices) of low-load intermittent Ct detection using NAAT? Current study for the first time provides follow-up data including bacterial loads. Thereby, our findings highlight the need for critical re-evaluation of which treatment outcomes should be considered positive or negative (e.g. failure) and how to define and measure these. The debate on treatment outcomes is important for other sexually transmitted infections as well, such as *Neisseria gonorrhea*, for which a single test-of-cure is recommended (NAAT or culture), yet follow-up data are lacking. Future laboratory research in developing new diagnostic tools (e.g. assessing viability of Ct) would greatly increase our understanding of treatment failure and the implications for health care. For now, several clinical implications can be concluded from our results. First, we consider it inappropriate to recommend the current single test-of-cure approach after 3 weeks [4], [7], [18]. Multiple NAAT tests provide a better insight into the detection pattern and can exclude missing intermittent positive tests. However, this might be practically impossible in routine care due to expected test compliance, acceptability and cost problems, in particular as it is yet unclear what the clinical implications of detection are. Second, symptoms are not a useful indicator for clinical decision-making. Symptoms usually prompt clinicians to perform a test-of-cure but are commonly reported after treatment only when systematically evaluated and do not appear to be specifically associated with Ct detection after 3 weeks. Third, alternatives for the single-dose 1000 mg azithromycin regimen may be considered, although the evidence for alternatives is inconclusive. While some studies reported lower detection rates after doxycycline [16], no differences were identified in a recent randomized controlled trial in men with non-gonococcal urethritis [17]. In 2010, the Netherlands changed the recommended treatment for anorectal Ct to doxycycline 2 dd 100 mg for 7 days. New options include delayed release, which requires fewer doses [26]. Fourth, in treated couples, a longer safe sex period might be advisable, although the recommended duration is uncertain, and compliance may be low. Fifth, re-testing for re-infection is recommended, thereby allowing substantial time between treating and re-testing (6–12 months) to capture established re-infections rather than transient detection events [2], [3]. Currently, only one-third of treated patients seen by general practitioners, specialists and STI clinics are re-tested within 6–12 months [27].

In conclusion, antimicrobial detection rates in azithromycin-treated Ct have been underestimated. Further study is needed to understand low Ct concentration implications (and, thus, the NAAT implications) in clinical patient treatment and testing management. Specifically, a debate is encouraged on the criteria on which to define and assess treatment outcomes.
